# Assessment of Masticatory Muscle Function in Patients with Bilateral Complete Cleft Lip and Palate and Posterior Crossbite by means of Electromyography

**DOI:** 10.1155/2020/8828006

**Published:** 2020-08-24

**Authors:** Liliana Szyszka-Sommerfeld, Agata Budzyńska, Mariusz Lipski, Sławomir Kulesza, Krzysztof Woźniak

**Affiliations:** ^1^Department of Orthodontics, Pomeranian Medical University in Szczecin, Al. Powstańców Wlkp. 72, 70111 Szczecin, Poland; ^2^Department of Preclinical Conservative Dentistry and Preclinical Endodontics, Pomeranian Medical University in Szczecin, Al. Powstańców Wlkp. 72, 70111 Szczecin, Poland; ^3^Department of Relativistic Physics, Faculty of Mathematics and Computer Science, Warmia and Mazury University in Olsztyn, Słoneczna Street 54, 10710 Olsztyn, Poland

## Abstract

**Aim:**

The aim of this study was to evaluate the electrical activity of the masticatory muscles in children with a bilateral complete cleft lip and palate (BCCLP) and posterior crossbite as well as in noncleft subjects with no malocclusion. Another purpose of the study was to examine the possible factors associated with this muscle activity.

**Methods:**

The study included 52 children with mixed dentition and Class I occlusions (20 patients with nonsyndromic BCCLP and 32 subjects with no clefts). All the cleft patients had posterior crossbite. The surface electromyography (sEMG) was used to identify the electrical potentials of the temporalis and masseter muscles. The electromyographical (EMG) recordings were taken with a DAB-Bluetooth Instrument (zebris Medical GmbH, Germany) at rest and during maximum voluntary clenching (MVC). The relationships between muscle EMG activity and independent variables were identified through multivariate logistic regression analysis.

**Results:**

The EMG activity of the temporalis muscles at rest was significantly higher in BCCLP patients with malocclusion in comparison with the noncleft subjects with normal occlusion. During MVC, significantly lower electrical potentials of the temporalis and masseter muscles were observed in cleft patients compared to the noncleft group. The presence of BCCLP, unilateral posterior crossbites, increased vertical overlap, and increased overjet are factors strongly associated with higher temporalis muscle EMG activity at rest.

**Conclusion:**

The use of surface electromyography in imaging muscle function showed that children with BCCLP and posterior crossbite exhibited altered masticatory muscle potentials at rest and during clenching. The presence of unilateral posterior crossbites, increased vertical overlap, and increased overjet had a significant impact on temporalis muscle activity in cleft patients. This knowledge is important in the aspect of early and proper diagnosis and orthodontic treatment of malocclusions, thereby achieving correct occlusion and improvement in muscle function.

## 1. Introduction

Clefts of the lip, alveolus, and/or palate are the most frequent congenital maxillofacial malformations that need multidisciplinary surgical and nonsurgical care performed from birth through to adulthood. Patients with cleft lip and palate (CLP) often suffer from aesthetic, morphological, and functional problems in the dentofacial region, in particular those concerning face aesthetics and masticatory function [1–3].

Malocclusions, such as anterior and/or posterior crossbite, often occur in cleft subjects because the development of the maxilla in CLP patients who have undergone surgery is frequently inhibited in the sagittal and transverse directions by scar tissue on the palate and the lip in contrast to those who have not undergone surgery [4]. Only proper occlusion can achieve balance within the stomatognathic system. A harmonized relationship between the dental arches is essential for maintaining functional symmetry. Moreover, improper occlusion may cause many functional problems, including severe temporomandibular disorders [5, 6]. In this way, patients with repaired CLP may exhibit disturbances in the stomatognathic system because of the presence of anterior and posterior crossbite [7]. Numerous studies have demonstrated the influence of malocclusions on masticatory muscle functioning in healthy subjects [8–12]. To the authors' knowledge, only a few studies have been conducted on the masticatory muscle activity in patients with CLP and malocclusions [13–15]. However, no reports have focused on bilateral cleft lip and palate (BCLP) patients. As a consequence, research on masticatory muscle function in bilateral cleft subjects is needed.

The assessment of the function of the stomatognathic system elements should be based on a detailed and scrupulous clinical examination. Nowadays, the progress in the development of various imaging techniques expands the diagnostic possibilities within the masticatory organ [16, 17]. The traditional clinical examination can be extended with measurable instrumental diagnostic methods that allow evaluating masticatory muscle functioning in an objective and quantitative way, such as electromyography (EMG), pressure algometry, or thermography [16–19]. One of the few diagnostic tools ensuring the most objective and precise imaging of muscle function and efficiency by identifying their electrical potentials is surface electromyography (sEMG). It makes it possible to assess the extent and duration of muscle activity. sEMG could be used as an additional electronic method in the quantitative analysis of muscle function in dentistry [19]. As sEMG identifies superimposed motor unit action potentials from many muscle fibers in terms of detecting surface electrodes, the analysis of EMG recordings is restricted to an assessment of general muscle activity, cooperation between different muscles, and variability in their activity over time. Another shortcoming of sEMG is its sensitivity to imbalances in impedance and this may reduce the accuracy of EMG measurements and as a consequence result in poor reproducibility. To ensure that this method is reproducible, the interelectrode distance should be fixed and a standard procedure for positioning the electrodes should be used so as to exclude variability in electrode placement. The inconsistency in impedance and reliability of sEMG could also be solved by the adequate quantitative analysis of the data with normalization procedures [19–21]. The most important advantage of sEMG is its noninvasiveness [19, 22]. Due to the simplicity of this method, its safeness, and availability, it has been used in studies on children [15, 17, 23–25].

The aim of this study was to determine whether the EMG activity of the masticatory muscles in children with bilateral complete cleft lip and palate (BCCLP) and posterior crossbites differs from that observed in noncleft individuals without malocclusion. We hypothesized that no differences exist between the cleft and noncleft patients with regard to the electrical potentials of the temporalis and masseter muscles at rest and during maximum clenching. Another purpose of the study was to identify the possible factors associated with EMG activity of the masticatory muscles.

## 2. Materials and Methods

The study protocol was approved by the Local Bioethics Committee of the Pomeranian Medical University and assigned the number KB-0012/08/15. Parental written informed consent was obtained for examination of children prior to the procedures and only consenting subjects were included.

Fifty-two children with mixed dentition and Class I occlusions were recruited to the study. The study population was divided into two groups: a cleft group comprising 20 children (10 girls and 10 boys) aged between 7 and 12 years (mean age 9.5 ± 2.3) with nonsyndromic bilateral complete cleft lip and palate and posterior crossbite, as well as a control group made up of 32 patients (15 girls and 17 boys) aged between 7 and 11 years (mean age 8.9 ± 1.5) with no cleft lip and palate and malocclusion. The cleft children were selected from a total of 70 patients who had been referred to the Cleft Care Centre in Szczecin, Poland, between April and June 2017. All had undergone lip and palate surgical reconstruction based on a similar surgical method and protocol, which was as follows: a two-stage lip closure at the age of about 3–6 months, followed by palate closure (hard and soft palate in one step) at the age of about 12 months. Excluded from the study were children with syndromic CLP, unilateral CLP, Class II or Class III malocclusions, dental caries, general muscle disorders, such as temporomandibular dysfunction (TMD), bruxism, history of neuromuscular disease, or disease affecting neuromuscular performance and children without lip and palate surgical repair. The application of the adopted inclusion and exclusion criteria resulted in 50 of the cleft subjects being excluded from the study and 20 of the participants being qualified for further examination.

The control group consisted of noncleft patients with no malocclusion, with normal lip seal, and with no previous orthodontic treatment. They were selected from children who had been referred to the Department of Orthodontics in Szczecin, Poland, between April and June 2017. The exclusion criteria also included the presence of Class II or Class III occlusions, dental caries, and general disorders affecting the muscles.

The function of the masticatory motor system was assessed on the basis of a clinical examination and electromyographic procedures. In the first part of the patient's examination, the general medical history of the patients was taken. This included information on any lip and palate surgery a child had undergone, such as the center where the cleft surgery was performed, the operating method, and the timing and sequence of the surgical repair as well as details on the masticatory motor system. During the next part, the patient's facial features were assessed. The intraoral examination consisted of an analysis of the dental arch shape on three planes and a mutual analysis of the upper and lower arches.

Following a conventional clinical assessment, electromyographic recordings of the masticatory muscles were taken. These methods had been previously employed by Szyszka-Sommerfeld et al. [15, 17, 25]. The EMG examination required each patient to sit in the standard position in a dental chair without any head support. Each subject was requested to assume a natural head position that avoided any undesired inclinations of the head [26]. EMG recordings of all the study subjects were taken by a single experienced examiner using a DAB-Bluetooth Instrument (zebris Medical GmbH, Germany). This electronic device is constructed in the Bluetooth Standard v1.2 short-range wireless communication technology in the 2.4 GHz ISM band. The DAB-Bluetooth Instrument consisted of a central unit, electrode cables, disposable electrodes, and a portable personal computer. The main component of the device was an 8-channel analog-to-digital converter. High- and low-pass filters were fitted to the sampling system input so as to limit the recorded signal frequency to the range of 4–310 Hz. The measuring system was equipped with active leads for 1.45 m long electrodes featuring preamplifiers with a symmetrical (differential) input with a variable gain level of 1000, 2500, and 5000 times. The use of two electrodes with similar impedance made it possible to achieve a high common-mode rejection ratio (CMMR) at a level of 110 dB. The cable ends were equipped with convenient automatic clamps for disposable electrodes [27].

During the study, the channels of the DAB-Bluetooth device were used with simultaneous acquisition for the sEMG recording, common grounding to all channels, high-pass filter of 7 Hz–5 kHz, channel sampling frequency of 1 kHz, converter dynamic resolution range of 12 bits, input impedance for the analog channels of 146 kΩ, and gain level of 1000 times. Disposable, self-adhesive silver/silver chloride (Ag/AgCl) bipolar surface electrodes with a fixed interelectrode distance of 20 mm (Noraxon Dual Electrode, Noraxon, USA) according to the project of SENIAM (Surface Electromyography for the NonInvasive Assessment of Muscle) were placed on the right and left anterior temporalis and the superficial masseter muscles parallel to the muscular fibers. In the case of the anterior temporalis muscle, the electrodes were positioned vertically along the anterior margin of the muscle, whereas in the case of the masseter muscle, they were parallel to the muscular fibers with the upper pole of the electrode at the intersection between the tragus-labial commissure and exocanthion-gonion lines [28]. A reference electrode was located inferior and posterior to the right ear.

Prior to the EMG examination, the surface of the patient's skin was cleaned with a 70% ethyl alcohol solution to reduce impedance. After the procedure, an impedance test was performed with a Metex P-10 measuring device (Metex Instruments Corporation, Korea) with an accuracy of 2% in a 4 × 10^2^–4 × 10^7^ Ω range to confirm that the examined area had been correctly prepared. This device measures the resistance between a pair of electrodes for a period of 5 minutes from the placing of the electrodes. Further examinations would be conducted if the test produced a positive result (low skin tissue impedance). The impedance of skin, targeting 1 × 10^3^–30 × 10^3^ Ω, was a condition, which had to be met in order to initiate the examination.

Before taking the EMG recordings, the patients were given instructions and asked to practice the necessary movements by copying the researcher. The recordings began 5 minutes later. The electrical activity of the temporalis and masseter muscles was measured in the clinical rest mandibular position and during maximum voluntary clench (MVC): (a) clenching as hard as possible in the intercuspal position for 5 seconds; (b) maximum clenching for 5 seconds with control substance that is with two 10 mm cotton rolls placed on the mandibular second premolar and molars or on the mandibular second milk molar and the first permanent molar.

Each activity was repeated at least three times and the EMG values obtained during the last two EMG measurements were averaged. The data obtained from the recordings were processed and graphically recorded. The EMG signal was amplified, digitized, and digitally filtered. An essential step in the initial processing of raw data to ensure reliable further analysis was the normalization process. Normalization involved relating the electrical potentials of the muscles to the reference values obtained from the EMG measurements performed during standardization recordings, which is maximum voluntary clenching with a control substance (cotton rolls placed on the mandibular teeth). Finally, the mean values of the EMG potentials (raw data) of the masticatory muscles measured both at rest and during MVC were expressed as a percentage of the mean electrical potentials (reference values) measured during the standardization test (clenching on the cotton rolls) according to the following formula: mean values (*μ*V) at rest or during MVC/mean values (*μ*V) during MVC on two cotton rolls × 100%. The EMG measurements presented in normalized values (unit *μ*V/*μ*V%) helped provide information on the impact of occlusion on neuromuscular activity, while avoiding individual anatomical and technical variability, such as electrode position, skin, and electrode impedance [29].

The asymmetry between the electrical activity of the left and right masticatory muscles was quantified by the Asymmetry Index (As, unit %, range from 0% to 100%), according to the following equation [30]:

(1) $$\begin{array}{c} \text{As}=\frac{\sum_{i=1}^{N}\left|R_{i}-L_{i}\right|}{\sum_{i=1}^{N}\left|R_{i}+L_{i}\right|}\times 100. \end{array}$$

The homogeneity of variance was evaluated using the Levene test. The normality was assessed using the Kolmogorov-Smirnov test. After checking that a set of data was normally distributed, Student's *t*-test or analysis of variance (ANOVA) was used to statistically analyze the results of the EMG recordings. The level of significance was set at 5% for all statistical analyses. The cut-off values for the continuous variables for the BCCLP group in relation to the noncleft group were determined by means of receiver operating characteristic (ROC) curves analysis. The obtained results were reported using the area under the curve (AUC), standard error (SE) of AUC, *P* value, and the ROC coordinate curves, that is, sensitivity and specificity. The relationships between muscle EMG activity and independent variables were identified through multivariate logistic regression analysis. The results were described by the odds ratio (OR), a 95% confidence interval (CI), and the *P* value.

## 3. Results

The normalized EMG activity of the temporalis and masseter muscles at rest and during MVC for both groups is presented in Tables 1 and 2. Temporalis muscle rest activity was significantly higher in subjects with BCCLP and posterior crossbite compared to the noncleft children with no malocclusion (*P*=0.021). Considerable disparities in the electrical activity of the temporalis muscles at rest were observed between girls with BCCLP and noncleft girls (*P*=0.028). There were no significant differences in the masseter muscle EMG potentials at rest between bilateral cleft patients and subjects with no cleft (*P*=0.890) (Table 1).

During clenching, significantly lower EMG potentials of the temporalis and masseter muscles were observed in BCCLP patients with malocclusion compared to the noncleft group with no posterior crossbite (for the temporalis muscles, *P*=0.030; for the masseter muscles, *P*=0.041) (Table 2).

The Asymmetry Index for the temporalis and masseter muscles at rest and during MVC was similar in both subjects with clefts and children with no clefts (*P* > 0.05) (Tables 1 and 2).

The analysis of the ROC curves exhibited that the greatest diagnostic efficacy in electromyography was achieved by such estimators of the distribution of variables as temporalis muscle activity at rest: AUC = 0.659, SE = 0.080, *P*=0.029, sensitivity = 45%, specificity = 91%, and cut-off point = 7.68 *μ*V/*μ*V%. According to multivariate logistic regression analysis, a strong association was observed between such factors as the presence of a BCCLP (*P*=0.012) and a unilateral posterior crossbite (*P*=0.003), as well as increased vertical overlap ≥ 3 mm (*P*=0.003), increased overjet ≥ 3 mm (*P*=0.003), and higher temporalis muscle EMG potentials (≥7.68 *μ*V/*μ*V%) during a resting position (Table 3).

## 4. Discussion

In this study, we evaluated masticatory muscle function in children with BCCLP and posterior crossbite by means of surface electromyography. Our analysis covered only individuals with bilateral CLP operated on for cleft based on a similar surgical protocol with a limited number of surgeons involved. All the children recruited to the study had Class I occlusions, thereby ensuring homogeneity of the sample and reducing the number of interfering factors.

Surface electromyography is an element in the quantitative assessment of patients in dentistry and the most objective and reliable diagnostic tool that allows imaging of muscle function by detecting their electrical potentials, thus determining the diagnosis and effectiveness of the orthodontic procedures applied [19, 23]. In children, sEMG is commonly performed because it is a noninvasive and an easy way of monitoring muscle activity through the use of surface electrodes located on the surface of the skin instead of a needle or fine wires which are used in the intramuscular electromyography [19, 22, 23]. However, it should be noted that applying of surface electrodes in cleft patients has some drawbacks. The principal shortcoming of this method is that the signal emitted by the surface electrodes may show not only the underlying muscle activity but also the surrounding connective tissue, skin thickness, and action potentials of nearby occurring nerves. This environment may have been different in cleft patients and may have been accounted for the observed variations.

We demonstrated that masticatory muscle EMG activity varied between the cleft and noncleft groups. Our experiment shows that, in children with BCCLP and posterior crossbite, the electrical activity of the temporalis muscles at rest was significantly greater compared with the normocclusive noncleft patients while temporalis and masseter muscle electrical potentials when clenching were much lower. According to Riise [31], the hyperfunction of the temporalis muscles at rest is a result of the existence of occlusal interferences and this may disturb the balance within the stomatognathic system. Higher temporalis rest activity may also suggest that there was muscle strain in that position [14]. Minimal electromyographic activity of the temporalis muscles observed in children with no cleft and malocclusion may indicate harmony between elevator and depressor muscles of the mandible in the rest position [32].

The reduced electrical potentials of the masticatory muscles observed in patients with BCCLP and posterior crossbite during MVC would suggest that a lower bite force is to be expected. According to Helkimo [33], masticatory muscle activity during maximum effort depends on the number of posterior occlusal contacts. The author suggested that a higher number of posterior contacts ensure stable intercuspal support and allow the elevator muscles to achieve greater muscle potentials during clenching or chewing. The decreased occlusal contact in patients with bilateral clefts and posterior crossbites may have reduced stimulation from the periodontal receptors and this may lead to the reduction of muscle activity. As muscle forces are directed to minimize joint loads and muscular efforts as a normal protective control [34], this hypofunction of the masticatory muscles during clenching may also be considered as an effective mechanism of protection in the stomatognathic system.

In our study, we also identified the factors that affect the altered masticatory muscle EMG activity. In the rest position, we found a strong association between increased temporalis muscle EMG potentials and such factors as the presence of a bilateral cleft, a unilateral posterior crossbite, increased vertical overlap, and increased overjet. However, these results should be interpreted with caution because of the small number of children involved, while also bearing in mind that a further study would be needed to verify the results.

As no studies on masticatory muscle function have been carried out on patients with bilateral CLP, it is difficult to compare our results with any others. Moreover, some earlier studies did not include sEMG signal normalization. This procedure was essential for the preliminary processing of raw data to ensure intercomparisons and further analysis, thereby removing most of the biological and technical variability [20]. In our report for data standardization purposes, the clenching test with two cotton rolls positioned on the mandibular molars was performed [28]. Nevertheless, a few studies involving electromyographic imaging of masticatory function have been conducted on patients with unilateral cleft lip and palate (UCLP). In a recent study, Szyszka-Sommerfeld et al. [15] analyzed the electrical potentials of the temporalis and masseter muscles in patients ranging in age from 6 to 13 years with unilateral complete cleft lips and palates (UCCLP), as well as in noncleft subjects. Seventy percent of the cleft children had posterior crossbites. The authors found that patients with unilateral CLP showed a significant increase in temporalis muscle activity at rest compared with the noncleft controls. They suggested that the presence of posterior crossbites affects temporalis muscle activity in cleft subjects. Li et al. [14] evaluated masticatory muscle function in patients aged 11–21 years with unilateral cleft lip and palate and anterior crossbite. Almost 90% of the cleft participants had posterior crossbites. The authors stated that compared to noncleft normocclusive controls UCLP subjects had significantly higher temporalis rest EMG activity and markedly lower electrical potentials of the masseter muscles during maximum clenching in the intercuspal position. In contrast to our own study, Li et al. observed greater masseter muscle activity at rest in subjects with CLP. An analysis of masticatory muscle function by means of electromyography in patients with unilateral complete cleft lip and palate was also the subject of a study conducted by da Costa et al. [13]. They investigated differences in the masticatory muscle electrical activity in children with UCCLP and children with no cleft ranging in age from 6 to 12 years. They found significantly higher EMG potentials of the temporalis and masseter muscles in CLP subjects at rest and during inactive period of mastication, while the electrical activity in the left masseter muscle and temporalis muscles during active period of mastication and in every muscle during isometry was much lower. The authors also observed that the muscles of cleft children remained active for longer than in noncleft children. Da Costa et al. suggested that the higher length of the masticatory cycle in cleft patients might be a consequence of malocclusion and might result in functional inefficiency, making mastication more difficult.

In summary, in the above-mentioned studies as well as in our study, the sEMG was used to identify masticatory muscle function in children and adolescents with CLP and abnormal occlusion. These reports proved that the alterations in masticatory muscle EMG activity exist between CLP and noncleft patients. The knowledge about the impact of the malocclusions on the alterations of masticatory muscle electrical potentials in cleft children is important in the aspect of early diagnosis and orthodontic treatment of malocclusions, thereby achieving proper occlusion and improvement in muscle function.

## 5. Conclusions

An analysis of the sEMG recordings revealed that children with bilateral cleft lip and palate and posterior crossbite had altered masticatory muscle electrical potentials at rest and during clenching in comparison to the patients with no cleft and normal occlusion. The presence of unilateral posterior crossbites as well as increased vertical overlap and increased overjet had a strong impact on temporalis muscle EMG activity in cleft subjects. In this way, proper and early diagnosis and orthodontic treatment of malocclusions is essential to achieve correct occlusion and improvement in muscle function. The surface electromyography is a useful diagnostic tool in imaging muscle function by detecting their electrical potentials in cleft children with malocclusion.

## Figures and Tables

**Table 1:** The results of the masticatory muscle EMG recordings at rest in the children studied.

Region	Variable	Gender	BCCLP group			Noncleft group			*P* value
			*n*	Mean	SD	*n*	Mean	SD	
Temporalis muscles	Electrical activity (*μ*V/*μ*V%)	Females	10	6.54	1.47	15	5.19	1.37	0.028^*∗*^
		Males	10	7.26	3.09	17	6.10	1.16	0.173
		Total	20	6.90	2.38	32	5.67	1.32	0.021^*∗*^
	Asymmetry Index (%)	Females	10	13.61	9.51	15	12.12	7.58	0.712
		Males	10	14.85	10.63	17	12.35	8.90	0.568
		Total	20	14.23	11.32	32	12.24	9.34	0.495

Masseter muscles	Electrical activity (*μ*V/*μ*V%)	Females	10	4.70	1.51	15	4.76	2.58	0.943
		Males	10	5.38	2.84	17	5.10	2.59	0.795
		Total	20	5.04	2.24	32	4.94	2.55	0.890
	Asymmetry Index (%)	Females	10	13.66	9.98	15	16.10	11.90	0.670
		Males	10	16.24	11.19	17	13.54	9.48	0.579
		Total	20	14.95	11.36	32	14.74	10.51	0.954

^*∗*^Statistically significant difference.

**Table 2:** The results of the masticatory muscle EMG recordings during clenching in the children studied.

Region	Variable	Gender	BCCLP group			Noncleft group			*P* value
			*n*	Mean	SD	*n*	Mean	SD	
Temporalis muscles	Electrical activity (*μ*V/*μ*V%)	Females	10	102.17	28.43	15	119.79	33.34	0.184
		Males	10	103.01	21.63	17	132.15	51.08	0.101
		Total	20	102.59	24.59	32	126.36	43.45	0.030^*∗*^
	Asymmetry Index (%)	Females	10	6.51	5.20	15	4.95	3.96	0.403
		Males	10	5.32	4.12	17	6.52	3.79	0.535
		Total	20	5.91	4.56	32	5.78	3.89	0.919

Masseter muscles	Electrical activity (*μ*V/*μ*V%)	Females	10	96.11	25.24	15	113.12	32.62	0.178
		Males	10	103.33	20.77	17	125.16	42.62	0.144
		Total	20	99.72	22.80	32	119.51	38.15	0.041^*∗*^
	Asymmetry Index (%)	Females	10	9.39	5.53	15	9.08	7.33	0.910
		Males	10	4.37	3.15	17	7.25	4.83	0.128
		Total	20	6.88	5.41	32	8.11	6.10	0.464

^*∗*^Statistically significant difference.

**Table 3:** Multivariate logistic regression analysis for children with increased temporalis muscle EMG potentials at rest (≥ 7.68 *μ*V/*μ*V%).

Variable	*n* (%)	OR	95% CI	*P* value
Bilateral cleft				
No (*n* = 32) (ref)	1 (3.1)	1.00		
Yes (*n* = 20)	7 (35.0)	20.15	1.92–171.96	0.012^*∗*^

Posterior crossbite				
No (*n* = 32) (ref)	1 (3.1)	1.00		
Unilateral (*n* = 6)	4 (66.7)	21.00	2.89–152.58	0.003^*∗*^
Bilateral (*n* = 14)	3 (21.4)	1.80	0.37–8.79	0.468

Vertical overlap				
0–3 mm (*n* = 37) (ref)	2 (5.4)	1.00		
≥ 3 mm (*n* = 6)	4 (66.7)	21.00	2.89–152.58	0.003^*∗*^
Reverse (*n* = 9)	2 (22.2)	1.76	0.29–10.58	0.536

Overjet				
0–3 mm (*n* = 37) (ref)	2 (5.4)	1.00		
≥ 3 mm (*n* = 6)	4 (66.7)	21.00	2.89–152.58	0.003^*∗*^
Negative (*n* = 9)	2 (22.2)	1.76	0.29–10.58	0.536

Lateral open bite				
No (*n* = 45) (ref)	6 (13.3)	1.00		
Yes (*n* = 7)	2 (28.6)	2.60	0.41–16.56	0.312

OR: odds ratio; CI: confidence interval; ref: reference category; ^*∗*^statistically significant difference.

## Data Availability

The datasets used to support the conclusions of this article are included within the article. Access to other data will be considered by the corresponding author upon request.
